# Retroperitoneal Cystic Lymphangioma: A Diagnostic and Surgical Challenge

**DOI:** 10.1155/2013/292053

**Published:** 2013-02-28

**Authors:** Oguzhan Güven Gümüştaş, Murat Sanal, Osman Güner, Volkan Tümay

**Affiliations:** ^1^Department of Radiology, Acıbadem Bursa Hospital, FSM Street, 16110 Bursa, Turkey; ^2^Department of Pediatric Surgery, Acıbadem Bursa Hospital, FSM Street, 16110 Bursa, Turkey; ^3^Department of Surgery, Acıbadem Bursa Hospital, FSM Street, 16110 Bursa, Turkey

## Abstract

A lymphangioma is a benign proliferation of lymph vessels, producing fluid-filled cysts that result from a blockage of the lymphatic system. The incidence of abdominal lymphangiomas is unknown; however they account for from 3% to 9.2% of all pediatric lymphangiomas, with retroperitoneal lymphangioma representing less than 1% of abdominal lymphangiomas. Due to rarity, preoperative diagnosis is often difficult.

## 1. Background

The incidence of abdominal lymphangiomas is unknown: however they account for from 3% to 9.2% of all pediatric lymphangiomas, with retroperitoneal lymphangioma representing less than 1% of abdominal lymphangiomas [1]. Although retroperitoneal lymphangiomas may sometimes be asymptomatic, they usually present as a palpable abdominal mass and are easily confused with other retroperitoneal cystic tumors including those arising from the liver, kidney and pancreas [2]. Retroperitoneal cystic lymphangiomas can present as a soft, slowly growing and painless mass. The mass may be an incidental finding during the evaluation of an unrelated complaint. Symptoms occur only after the enlarging mass distorts and compresses the adjacent structures (i.e., partial intestinal obstruction, ureteric obstruction) [3]. They may become symptomatic if they become large enough to impose on surrounding structures. Retroperitoneal lymphangiomas manifest with clinical symptoms of abdominal pain, fever, fatigue, weight loss, and hematuria, due to their size and occasionally might be complicated by intracystic hemorrhage, cyst rupture, volvulus or infection [4, 5]. Differentiating cystic lymphangiomas from other cystic growths by imaging studies alone is often inconclusive and surgery is most frequently required for definitive diagnosis and to ameliorate the symptoms [6]. Cystic retroperitoneal lymphangioma in children is a difficult and important diagnosis. Because of this reason we are reporting a case of retroperitoneal cystic lymphangioma.

## 2. Case Report

**Figure 1:**
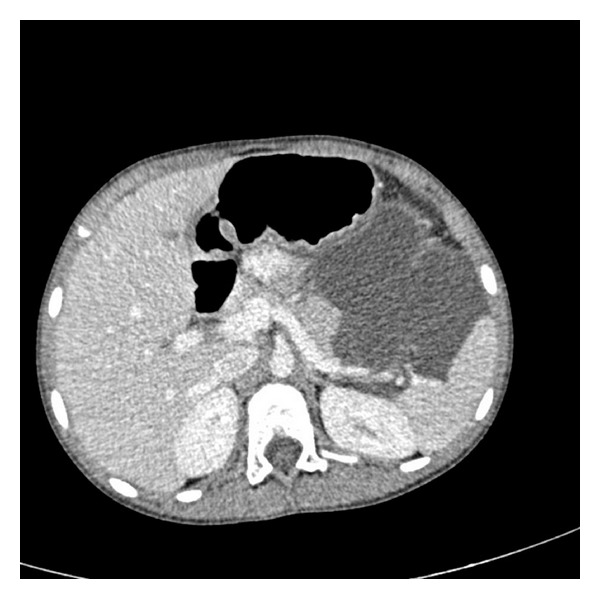
Contrast-enhanced CT axial scan shows retroperitoneal lobulated-septated cystic mass between spleen, stomach, and pancreas. Splenic vein and artery borders are in the cystic mass. Also cystic mass reaches to the pararenal space.

**Figure 2:**
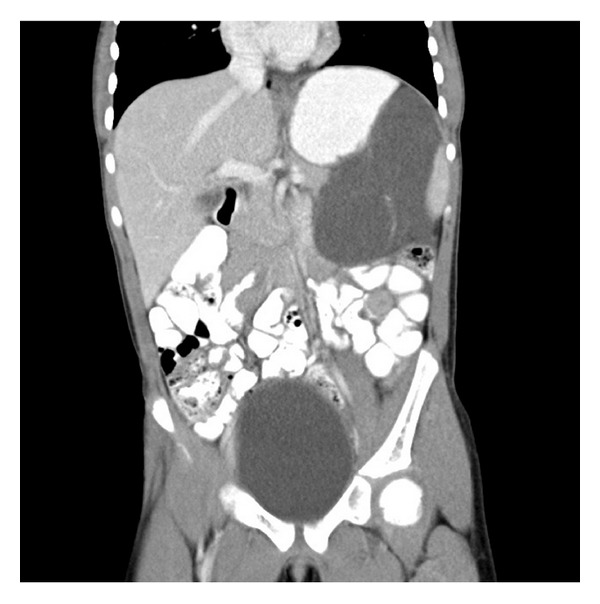
Contrast-enhanced CT coronal scan shows retroperitoneal lobulated-septated cystic mass that reaches to subdiaphragmatic space.

**Figure 3:**
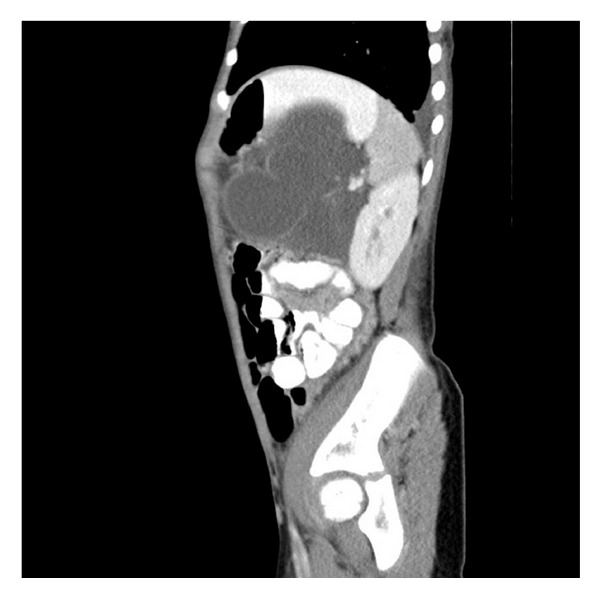
Contrast-enhanced CT sagital scan shows that retroperitoneal lobulated-septated cystic mass cannot be distinguished from spleen, pancreas, and stomach. Splenic vein and artery borders are in the cystic mass also.

A 8-years-old girl presented to the emergency room with abdominal pain, nausea, and vomiting at another hospital. Ultrasonography of the abdomen revealed a thin-walled, multiloculated, anechoic cystic mass with septations occupying almost left quadrant of the abdomen. The initial diagnosis was mesenteric cyst. One day after the patient has consulted at our hospital. On contrast-enhanced CT (Figures 1, 2, and 3) was a cystic septate mass, which borders cannot be distinguished from spleen, pancreas, and stomach.

After stabilisation of the child midline laparotomy was performed and there was neither intraabdominal located mass nor mesenteric cyst. 

Exploration revealed a retroperitoneal cystic structure beneath the stomach. Retroperitoneal space was explored, dividing gastrocolic ligament, and there was an approximately 20 × 15 cm cystic mass, which showed strong adhesions to the distal part of the pancreas, to the lower pole of the spleen and to the posterior surface of the stomach. Meticulous preparation of the mass was performed and complete excision was achieved without any partial resection of the associated organs. 

Pathology confirmed the diagnosis of the cystic lymphangioma. Postoperative course was uneventful and the child discharged on postoperative day 5.

## 3. Conclusion

**Figure 4:**
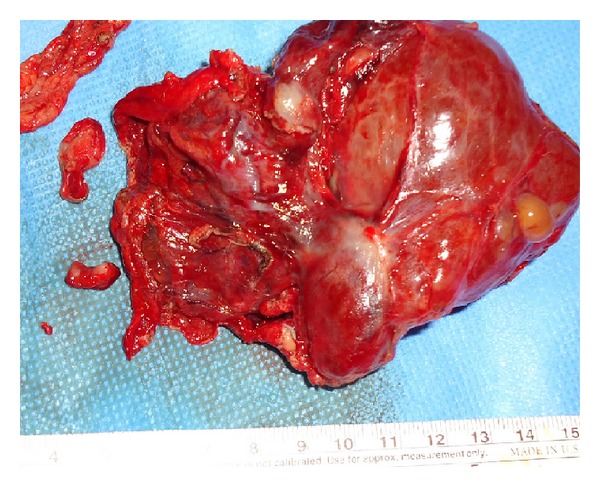
Total excision of the retroperitoneal lymphangioma.

 The lymphatic system is derived during the third or fourth fetal month from 2 paired and unpaired endothelial channels proliferate centrifugally from these sacs, which are located in the neck, mesenteric root, and bifurcation of the femoral and sciatic veins [3]. A lymphangioma is a benign proliferation of lymphatic tissue believed to originate from the early sequestration of lymphatic vessels that fail to establish connections with normal draining lymphatics. Lymphangiomas are therefore considered a congenital rather than an acquired tumor. After birth, they can become markedly dilated as a result of both the collection of fluid and the budding of preexisting spaces. They may form unilocular or multilocular cystic masses and can encroach on vital structures [3]. Upon pathologic examination, lymphangiomas are thin-walled cystic masses with a smooth gray, pink, or yellow external surface (Figure 4). 

On cut section, they vary in appearance and may contain large macroscopic interconnecting cysts often referred to as cystic hygroma or cystic lymphangioma or microscopic cysts cavernous lymphangioma [7]. The cysts may contain chylous, serous, hemorrhagic, or mixed fluid [8]. Histologically and dilated lymphatic spaces are lined with attenuated endothelial cells resembling the cells that line normal lymphatics. The lymphatic spaces are usually filled with proteinaceous eosinophilic fluid. The supporting stroma is composed of collagen and may contain lymphocytes and lymphoid aggregates [7]. Malignant degeneration to lymphangiosarcoma is rare in children and adolescents. Those in the retroperitoneal site are almost always of the cystic type [3]. The incidence of lymphangiomas is unknown. Most series observe no sex predilection. Most cystic lymphangiomas present within the first 2 years of life with 50% to 60% manifesting by age 1 year and 90% by age 2 years. The incidence of lymphangiomas occurring in the head and neck ranges from 50% to 75%. As many as 20% are found in the axilla. The extremities, mediastinum, lungs, bone, and abdominal viscera mesentery are additional sites for lymphangiomas [3]. Lymphangiomas of the retroperitoneum are usually diagnosed in older children or adults. The acute presentation of lymphangiomas can cause abdominal pain, tenderness, distension, fever, leukocytosis, peritonitis, dysuria, and guarding.

 Radiologic diagnosis of retroperitoneal masses is often made using ultrasound imaging or computed tomography (CT). Sonographically, lymphangiomas are most often multilocular cystic masses that are anechoic or contain echogenic debris. Intravenous contrast-enhanced CT may show enhancement of the cyst wall and septa [9]. The fluid component is typically homogeneous with low attenuation values. Occasionally, negative attenuation values occur in the presence of chyle. Calcification may occur but is uncommon [9]. If hemorrhage occurs, the intracystic attenuation values may simulate a solid tumor mass or abscess. The mass may traverse adjacent retroperitoneal anatomical compartments, displacing organs and vessels [3]. They can compress and infiltrate vital structures or present with complications like intracystic hemorrhage, cyst rupture, volvulus, or infection [10]. 

The sonographic appearance of a septated cystic mass with clear fluid is supposed to be a characteristic of a lymphangioma [11]. However, preoperative diagnosis is often difficult, in part because there is little to distinguish them from other cystic masses and because the lesion is often not considered on the differential diagnosis due to rarity.

Diagnostic important point for cystic lymphangioma: an elongated shape and a crossing from one retroperitoneal compartment to an adjacent one. Also at CT, cystic lymphangioma typically appears as a large, thin-walled, multiseptate cystic mass [12, 13]. A teratoma with a large cystic component can resemble a lymphangioma [3].

The treatment of choice is complete surgical excision. The long-term prognosis is excellent if complete excision has been achieved. If not the child should be followed up with serial ultrasound to exclude recurrence of the lesion. An alternative treatment available is image-guided percutaneous catheter drainage of lymphangioma followed by sclerotherapy [13].
